# Predicting 180-day mortality for women with ovarian cancer using machine learning and patient-reported outcome data

**DOI:** 10.1038/s41598-022-22614-1

**Published:** 2022-12-08

**Authors:** Chris J. Sidey-Gibbons, Charlotte Sun, Amy Schneider, Sheng-Chieh Lu, Karen Lu, Alexi Wright, Larissa Meyer

**Affiliations:** 1 Section of Patient-Centered Analytics, Department of Symptom Research, University of Texas MD Anderson Cancer Center, Houston, USA; 2 Department of Gynecologic Oncology and Reproductive Medicine, University of Texas MD Anderson Cancer Center, Houston, USA; 3 Department of Medical Oncology, Dana Farber Cancer Institute, Boston, USA; 4 Department of Medicine, Harvard Medical School, Boston, USA

**Keywords:** Ovarian cancer, Outcomes research

## Abstract

Contrary to national guidelines, women with ovarian cancer often receive treatment at the end of life, potentially due to the difficulty in accurately estimating prognosis. We trained machine learning algorithms to guide prognosis by predicting 180-day mortality for women with ovarian cancer using patient-reported outcomes (PRO) data. We collected data from a single academic cancer institution in the United States. Women completed biopsychosocial PRO measures every 90 days. We randomly partitioned our dataset into training and testing samples. We used synthetic minority oversampling to reduce class imbalance in the training dataset. We fitted training data to six machine learning algorithms and combined their classifications on the testing dataset into an unweighted voting ensemble. We assessed each algorithm's accuracy, sensitivity, specificity, and area under the receiver operating characteristic curve (AUROC) using testing data. We recruited 245 patients who completed 1319 PRO assessments. The final voting ensemble produced state-of-the-art results on the task of predicting 180-day mortality for ovarian cancer paitents (Accuracy = 0.79, Sensitivity = 0.71, Specificity = 0.80, AUROC = 0.76). The algorithm correctly identified 25 of the 35 women in the testing dataset who died within 180 days of assessment. Machine learning algorithms trained using PRO data offer encouraging performance in predicting whether a woman with ovarian cancer will die within 180 days. This model could be used to drive data-driven end-of-life care and address current shortcomings in care delivery. Our model demonstrates the potential of biopsychosocial PROM information to make substantial contributions to oncology prediction modeling. This model could inform clinical decision-making Future research is needed to validate these findings in a larger, more diverse sample.

## Introduction

Ovarian cancer is the most common cause of death for patients with gynecologic cancers in the United States, and it is responsible for 5% of cancer-related deaths in women overall^1^. More than 70% of patients with ovarian cancer are diagnosed with late-stage disease due to ineffective screening^2^. While nearly half of women diagnosed with ovarian cancer survive five years after diagnosis (47%), only 29% of those diagnosed with late-stage disease live that long^3^.

Despite initial response to chemotherapy, most patients with ovarian cancer experience disease recurrence and eventually develop chemoresistance to multiple lines of therapy^2^. Treatment for recurrent ovarian cancer seeks to maximize survival and quality of life (QoL). While cure rates have not improved significantly in recent years, there has been a notable prolongation of survival through the careful sequential use of drugs^4^. Many treatments can be associated with painful and distressing side effects, including neuropathy, mouth sores, nausea, vomiting, and fatigue which can severely reduce patient QoL^4^. For women with recurrent disease, chemotherapy inevitably becomes palliative rather than curative. There are often genuine tradeoffs between attempts to prolong survival and reduce symptoms while maintaining quality of life (QoL)^4^.

Although national guidelines recommend that intensive, hospital-based care be avoided at the end of life, 40–60% of women with recurrent ovarian cancer receive aggressive care near death^5–9^. Failure to meet guidelines for end-of-life (EoL) care reduces patient quality of life^10^. There is growing evidence that high-cost, high-intensity treatments delivered at the EoL are not associated with improved quality of life, quality of care, or medical outcomes^11,12^.

Research has shown that oncologists' tendency to overestimate survival drives, at least in part, the under-utilization of existing EoL services^13^. Christakiset and colleagues demonstrated that oncologists overestimate patient prognoses by a factor of five and are even less accurate when they have longstanding relationships with patients or frequent visits—both of which are true for ovarian cancer^11^. There is a critical need to support clinical decision-making by developing prediction tools that can reliably identify when a woman is nearing the EoL. These tools could empower clinicians and patients with the timely information needed to help patients make medical decisions congruent with their informed preferences.

Patient-reported outcome measures (PROMs) are standardized tools that allow patients to report on their wellbeing, health, and functioning. The data from PROMs is helpful to inform clinical practice as well as research and quality improvement initiatives^14,15^. While PROMs are increasingly collected to inform clinical care; PRO data are not well represented in many EHR systems^16^. Because PROMs can capture comprehensive indicators of patient health and wellbeing at frequent intervals and with high accuracy, we hypothesize that PRO data may be beneficial for developing robust prediction tools.

In this manuscript, we attempt to create a solution to the issue of poor prognostication around the end-of-life by using longitudinal PRO data to develop a novel ML algorithm to accurately and sensitively predict transition to end-of-life for women with ovarian cancer.

## Methods

We recruited patients from a single large academic cancer institution in the United States. After study enrollment, we collected baseline assessments were using electronic forms administered through REDCap electronic data capture software^17^. Thereafter, PROMS were administered longitudinally every 90 days until death or discharge to hospice. All participants provided written informed consent. Ethical approval was provided by the MD Anderson Institutional Review Board and all research was conducted in accordance with the Declaration of Helsinki.

We included six PROMs in our data collection, which measured symptom severity and interference (MDASI-OC)^17^, health status (EQ-5D, depression, and anxiety, using the CESD and GAD-7)^18,19^, and health-related quality of life (FACT-OC)^20^. A list of measure and their assessment time points is shown in Table 1.

**Table 1:** Study domains, measures, and assessment frequency.

Domain	Measure	Frequency
Demographic information		Baseline
Symptoms	MDASI-OC	Baseline, 90 days
Depression	CESD-20	Baseline, 90 days
Anxiety	GAD-7	Baseline, 90 days
Health-related quality of life	EQ5D-5L	Baseline, 90 days
Cancer-related quality of life	FACT-O	Baseline, 90 days

International guidelines were used to inform our algorithm development protocol^21^^,^^22^. We have used these techniques in prior research^23–25^. We used the Prediction Model Study Risk of Bias Assessment Tool (PROBAST) to help ensure the generalizability of our models^26^. Data were cleaned, centered, and normalized^27,28^. We created variables to represent the change in PROM scores between the current and baseline assessments. The entire dataset was randomly partitioned with a 2:1 ratio into training and testing datasets with stratification around the outcome variable to ensure equal proportions of events to non-events in both datasets. We used *k*-means Synthetic Minority Oversampling Technique (SMOTE) to oversample the cases within the minority class^29,30^. This approach has been shown to improve the performance of algorithms in class imbalanced datasets^31,32^. Creating synthetic data is associated with overfitting risk, which we mitigated using feature selection, cross-validation, and independent testing techniques discussed below. We did not apply SMOTE to the testing dataset. Other studies predicting discharge mortality following acute ischemic stroke have successfully utilized oversampling to create a 1:1 ratio of classes in their previously imbalanced training dataset^33^.

Missing data were imputed using multiple chained equations (MICE); fewer than 5% of data points were missing and deemed missing at random^34^. We did not impute data for the outcome variable.

### Outcome variable and performance metrics

Death within 180 days of an assessment was the predicted outcome variable. We reached a consensus that this time point was suitable for signaling a transition to the EoL and prompting productive EoL conversations. We decided that sensitivity, the ability to correctly identify women who will die within 180-days of assessment, was a key performance metric alongside area under the receiver operating characteristics curve (AUROC).

### ML models

We evaluated seven ML algorithms. We have experience using each of these models^23,35,36^. We have found that by combining several tools, it is possible to assess the relative strengths of the models in terms of their prediction power and gain unique insights into the variables driving model performance. We included algorithms that fall along a continuum from interpretable linear algorithms to more complex, and therefore less interpretable, nonlinear algorithms^23^.

#### Logistic regression with elastic net regression (GLM)

We used elastic net regularization, which combines Ridge and Least Absolute Shrinkage and Selection Operation (LASSO) techniques^37,38^. The hyperparameters were lambda (the degree of regularization) and alpha (the type of regularization where alpha = 1 is the LASSO and 0 is the ridge penalty and values in between represent the elastic net penalty.

#### General additive model (GAM) with spline smoothing

The GAM algorithm provides a nonlinear extension to logistic regression, allowing us to model more complex relationships within the data. The hyperparameter was degrees of freedom.

#### Regression trees (tree)

Regression trees create predictions by partitioning data into a series of decision nodes. The hyperparameters were the number of features to include and the maximum depth of the trees.

#### Gradient boosted trees (treeboost)

Gradient boosting trees expand on the regression tree algorithm by creating multiple trees which are sequentially developed to reduce the error across the training set. The hyperparameters were the number of trees to include, the number of features, and the maximum depth of each tree.

#### Multivariate adaptive regression splines (MARS)

The MARS algorithm can describe nonlinear interrelationships between features and automatically select only the most relevant features^39^. We evaluated hyperparameters, including number of prunes (the terms included in the final model) and the number of interactions allowed between variables.

#### Support vector machines (SVM)

Support vector machines utilize complex feature space transformation in order to apply a hyperplane to separate the different classes^40^. The utilization of the radial basis function allows complex nonlinear interactions to be modeled^23^. We assessed both 'C' (the penalty applied for each misclassified datapoint) and gamma (the curvature of the decision boundary) hyperparameters.

#### Neural networks (NN)

Neural networks are designed to mimic the features of the mammalian cortex^41^. They include an input layer, several hidden layers, and an output layer. Feature values are combined and modified using an activation function for complex nonlinearities within the data. The hyperparameters we assessed were the number of hidden layers and units within those layers.

We used tenfold cross-validation to develop models using the training data^42^. For all models, hyperparameters were optimized utilizing a random grid search^43^.

We combined the prediction from the individual algorithms described above into a final classification to derive our final predictions. We took the final classification, which most algorithms decided on. Numerous studies demonstrate that using an ensemble can reduce prediction error^44^. Studies have shown combined preprocessing (e.g., SMOTE) techniques and ensemble methods outperform preprocessing techniques alone on class imbalanced data^45^.

We opted to assess discrete binary predictions (i.e., event/no event) rather than probabilistic predictions (e.g., 80% mortality risk) from our models for three reasons. First, we used algorithms that are known to perform well in binary classification tasks but have issues with both over- and under-confidence when predicting continuous probabilities, such as neural networks and support vector machines^46^. Second, in our experience, calibration is negatively affected by oversampling. Third, combining poorly-calibrated probability predictions, rather than robust binary predictions, into an ensemble could reduce our predictions' final performance rather than improve them.

## Results

### Baseline and clinical characteristics

We show the baseline patient demographic information in Table 2. Overall, the 243 participants completed 1319 assessments (median 5 per patient), and 143 assessments were completed within 180 days of a patient dying.

**Table 2:** Baseline demographic characteristics of study participants.

	Mean	SD
Age	64	9
People in household	2	1
**Marital status**	**N**	**%**
Married/partnered	170	70
Divorced	33	14
Widowed	21	9
Seperated	1	0
Single, living alone	10	4
Never married	7	3
**Race**	**N**	**%**
American Indian/Alaskan native	1	0
Asian	8	3
Native Hawaiin or other Pacific Islander	1	0
Black or African American	21	9
White	203	84
Other	9	4
**Education**	**N**	**%**
Elementary or lower	7	3
High school non-graduate	32	13
High school graduate	56	23
College (1 year or more)	145	60
**Income**	**N**	**%**
Less than $25,000	20	10
$25,000–$34,999	9	4
$35,000–$49,999	28	13
$50,000–$74,999	19	9
Greater than $75,000	134	64

### Training and testing of algorithms

The accuracy, sensitivity, specificity and AUROC for the seven algorithms and the final ensemble are displayed in Table 3. Details of the final hyperparameters used for each model are available in the Supplementary Materials. Algorithms generally performed well across all performance metrics. Boosted trees displayed the highest accuracy (0.87) and specificity (0.94) but the poorest sensitivity (0.29). The neural network produced the highest sensitivity of 0.80, correctly identifying 25 of 35 women in the test dataset who died within 180-days of assessment. The confusion matrix for the final ensemble is shown in Table 4. The final ensemble had the most consistent performance across all metrics, with sensitivity = 0.71 and specificity = 0.80.

**Table 3:** Final model performance.

Algorithm	Acronym	Accuracy	Sensitivity	Specificity	AUROC
Generalized linear model	GLM	0.76	0.69	0.77	0.73
General additive model	GAM	0.80	0.62	0.87	0.71
Regression tree	Tree	0.85	0.34	0.90	0.62
Boosted trees	XGTree	0.87	0.29	0.94	0.61
Multivariate adaptive Regression splines	MARS	0.85	0.60	0.88	0.74
Support vector machine	SVM	0.68	0.74	0.67	0.71
Neural network	nnet	0.73	0.80	0.73	0.76
Final ensemble		0.79	0.74	0.75	0.76

**Table 4:** Confusion matrix for the final ensemble.

		Patient died within 90 days?	
		No	Yes
Ensemble prediction of 90-day mortality	No	63	5
	Yes	6	11

**Figure 1:**
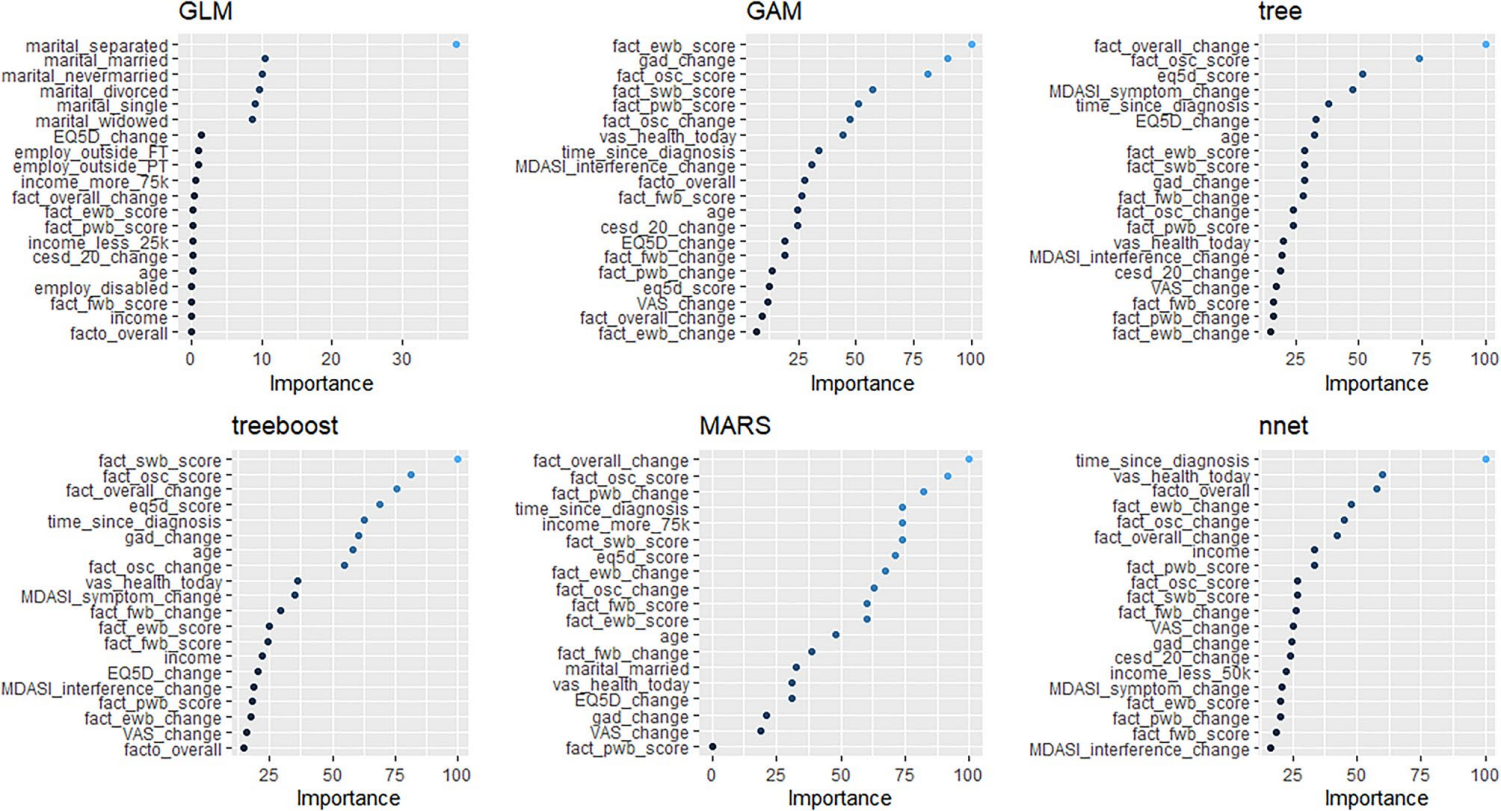
Variable importance plots.

The relative importance of the features in the model is shown in Fig. 1. The Figure demonstrates the importance of each variable within each model. The change in the FACT-O overall score was the most important variable in both the MARS and tree algorithm. The high representation of FACT subscales among the most important variables across algorithms demonstrates the value of HRQOL and psychosocial data in making mortality predictions.

## Discussion

Machine learning algorithms trained using PROM data offer state-of-the-art performance in predicting whether a woman with ovarian cancer will die within 180 days. We present a novel approach which combines longitudinal PRO data with ML techniques to achieve high performance and, in so doing, we highlight the importance of patient-reported data in ML models of mortality.

At present, the gold standard for prognostication depends upon individual physicians' assessments of clinical factors (e.g., cancer stage, performance status, response to prior treatments) and more nuanced assessments (e.g., past experiences taking care of similar patients). However, the subjective nature of these assessments frequently results in overly optimistic estimates that prevent physicians, patients, and family caregivers from making informed end-of-life decisions that are congruent with patients' preferences^47^. Several predictive tools have been previously developed (e.g. the Palliative Performance Index, Palliative Prognostic Score). While these measures have been validated in patients with advanced cancer, they remain dependent upon subjective assessments of the patients' functional status as a core component, without integrating any data from patients themselves. This subjective approach is error-prone and may underlie the fact that any women with ovarian cancer do not receive guideline-recommended care at the end of life.

Previous attempts have been made to develop end-of-life prediction models in oncology using EHR data. These studies have shown good overall performance on the task but have demonstrated very low sensitivity (< 0.30), indicating that the models were competent in predicting who would not die following an observation but, criticially, were not capable of reliable estimation of patients who would actually die. A systematic review conducted by our group found that most models developed to predict mortality for cancer patients suffered from high risk of bias relating to the manner in which the work was performed or reported^48^.

Our findings extend prior work by incorporating PRO data into data-driven ML models designed to predict 180-day mortality. Patient-reported outcome data has been widely praised for accurately reflecting patients' health and experience. In the current study we were able to accurately track patients' own reports of their symptoms, functioning, and QoL across multiple domains. These variables were highly prioritized by the ML models, with psychosocial elements of a patient's life, including emotional and social wellbeing, being among the most informative variables in many models. Interestingly, these psychosocial features were often more informative than changes in participants' physical health, symptom severity and interference, and functional status across models. These findings suggest that comprehensive patient-reported biopsychosocial information may provide key signal when deriving high-quality predictive models. Serious consideration should be given to collecting this data in initiatives seeking to develop similar models in other fields.

In terms of ML methodology, we endeavored to apply techniques that have been shown to improve the sensitivity of models trained on class-imbalanced data in other fields, such as financial risk prediction^49–52^. Without such techniques, the ML models are at risk of learning that they can achieve high accuracy across the entire dataset without ever correctly identifying the minority class (i.e., patients who die within 180-days of assessment). We were able to correctly identify most patients who died within 180-days of assessment. This represents a substantial improvement in performance compared to other generic oncology mortality algorithms, which have reported sensitivities below 0.30^53,54^. One limitation of the techniques which we have used to deal with class imbalance is that it is known to reduce a model's calibration when presenting a continuous probability. With this in mind, we decided to present models that made categorical predictions. Research to reduce the negative interaction between oversampling techniques and calibration error is ongoing and future iteration of these models may be capable of reliably producing continuous risk estimates^55^.

Though our models are highly sensitive, there were false positives. Our intention is that, in practice, this algorithm will be used to identify women who may be at higher risk of death to begin discussions about end-of-life care. The algorithm may be beneficial given one criterion for hospice enrollment is an estimated life expectancy of six months or less. There is much work to be done on the communication of mortality prediction results to patients. Still, we hypothesize that it may be easier to communicate results from predictions that are known to sometimes overestimate mortality risk than those which are known to be especially specific.

In the current study, we elected to us an ensemble of ML tools to generate the most robust predictions. There are some advantages and disadvantages of this process. We sought to use the ensemble methodology as a way of improving prediction quality and balancing out peculiarities of the individual models. To this end, we were successful; the ensemble produced the best overall performance across multiple metrics. One disadvantage of this approach is the difficulty in interpretating model predictions. Our rationale for accepting this disadvantage was that the many of the features we were included were considered to be reflective of transition to end-of-life rather than formative (e.g., quality of life and mental health). The purpose of the algorithm is not to identify areas amenable to intervention but rather to create a reliable prediction which can inform the correct timing of shared decision-making and end-of-life planning.

We acknowledge that our study has limitations. We used data from a single specialty cancer center with a preponderance of well-educated, white, and affluent women within our sample. While our study successfully demonstrated the utility of PRO data in the development of ML algorithms to predict mortality in this population and that our models appear to compare favorably to those developed using electronic health record (EHR) data alone; we were unable to make direct comparison between PRO and EHR data in our study. The addition of more diverse data including health record data may improve the quality of our predictions. Future studies are needed to evaluate the performance of these models in a more diverse group of women and compare the relative influence of different data sources, including PRO and EHR data on the development of high-quality models.

In conclusion, we demonstrate that state-of-the-art performance is achievable by developing ML models which utilize longitudinal PRO data as well as strategies for overcoming class imbalance. Our models performed especially well in terms osf sensitivity and were able to correctly identify most women who would die within 180-days of assessment. Adopting such models into clinical practice can inform end-of-life clinical decision making and improve utilization of guideline-recommended EoL services, including palliative care.

## Data Availability

The datasets analysed during the current study are not publicly available due to the inclusion of identifiable information but de-identified data required for the completion of specific analyses can be made available upon request.
